# Diplomas in Absentia Still in Demand

**Published:** 1894-01

**Authors:** 


### DIPLOMAS IN ABSENTIA STILL IN DEMAND.

THE following letter, with translation, indicates that the demand for diplomas in Germany has not ceased. These applications, while not as frequent as formerly, still demonstrate that the fraudulent diploma is still circulated, and must have a certain value to the recipient.

We publish this not on account of its rarity, but to call the attention of the State Board of Examiners of Wisconsin to the fact stated in the letter. The "Wisconsin Dental College" has been something of a myth for a number of years, and it would gratify many to have its history and work explained.

[*Vertraulich!*]

18 November, 1893.

JAMES TRUMAN, DEAN OF THE UNIVERSITY OF PENNSYLVANIA, DEPARTMENT OF DENTISTRY, 3243 Chestnut Street, Philadelphia:

HOCHGEEHRTER HERR PROFESSOR,—Frage hiermit ergebenst bei Ihnen an, ob Sie mir nicht das Diplom Ihres geschätzten College verleihen wollen.

Ich bin im Jahre 1889 von dem Wisconsin Dental College reputable als Doctor of Dental Surgery diplomirt und bin zur Einsendung jeder Examen Arbeit bereit. Wegen meiner Familie ist es mir nicht möglich nochmals hinüber zu kommen, und bitte ich Sie daher mir Ihre Bedingungen gefälligst *baldigst* mitzutheilen.

Im Voraus meinen verbindlichsten Dank, begrüsse ich Sie unter der Versicherung strengster Discretion.

TRANSLATION.

[*Confidential.*]

November 18, 1893.

JAMES TRUMAN, DEAN OF THE UNIVERSITY OF PENNSYLVANIA:

HIGHLY HONORED PROFESSOR,—I ask you whether you will be willing to invest me with the diploma of your greatly esteemed college.

I received the regular diploma of D.D.S. from the Wisconsin Dental College in 1889, and am ready to send in to you any examination work that you may request.

On account of my family it is impossible for me to cross the ocean again, and I beg you to send me the terms as quickly as possible.

I return in advance my thanks and salute you under the assurance of great dircretion.

